# Mentalizing and motivation neural function during social interactions in autism spectrum disorders^☆^

**DOI:** 10.1016/j.nicl.2013.09.005

**Published:** 2013-09-19

**Authors:** Michal Assaf, Christopher J. Hyatt, Christina G. Wong, Matthew R. Johnson, Robert T. Schultz, Talma Hendler, Godfrey D. Pearlson

**Affiliations:** a Olin Neuropsychiatry Research Center, Institute of Living, Hartford, CT, USA; b Department of Psychiatry, Yale University School of Medicine, New Haven, CT, USA; c Department of Neurobiology, Yale University School of Medicine, New Haven, CT, USA; d University of Nottingham, Malaysia Campus, Semenyih, Selangor, Malaysia; e Center for Autism Research, Children's Hospital of Philadelphia, Philadelphia, PA, USA; f Department of Pediatrics, Pereleman School of Medicine, University of Pennsylvania, Philadelphia, PA, USA; g Functional Brain Research Center, Wohl Institute for Advanced Imaging, Tel Aviv Sourasky Medical Center and Sackler Faculty of Medicine, Tel Aviv University, Israel

**Keywords:** Theory of mind, Reward, Nucleus accumbens, Middle temporal gyrus

## Abstract

Autism Spectrum Disorders (ASDs) are characterized by core deficits in social functions. Two theories have been suggested to explain these deficits: mind-blindness theory posits impaired mentalizing processes (i.e. decreased ability for establishing a representation of others' state of mind), while social motivation theory proposes that diminished reward value for social information leads to reduced social attention, social interactions, and social learning. Mentalizing and motivation are integral to typical social interactions, and neuroimaging evidence points to independent brain networks that support these processes in healthy individuals. However, the simultaneous function of these networks has not been explored in individuals with ASDs. We used a social, interactive fMRI task, the Domino game, to explore mentalizing- and motivation-related brain activation during a well-defined interval where participants respond to rewards or punishments (i.e. motivation) and concurrently process information about their opponent's potential next actions (i.e. mentalizing). Thirteen individuals with high-functioning ASDs, ages 12–24, and 14 healthy controls played fMRI Domino games against a computer-opponent and separately, what they were led to believe was a human-opponent. Results showed that while individuals with ASDs understood the game rules and played similarly to controls, they showed diminished neural activity during the human-opponent runs only (i.e. in a social context) in bilateral middle temporal gyrus (MTG) during mentalizing and right Nucleus Accumbens (NAcc) during reward-related motivation (P_cluster_ < 0.05 FWE). Importantly, deficits were not observed in these areas when playing against a computer-opponent or in areas related to motor and visual processes. These results demonstrate that while MTG and NAcc, which are critical structures in the mentalizing and motivation networks, respectively, activate normally in a non-social context, they fail to respond in an otherwise identical social context in ASD compared to controls. We discuss implications to both the mind-blindness and social motivation theories of ASD and the importance of social context in research and treatment protocols.

## Highlights

• We used an fMRI Domino game to map social networks in high-functioning ASDs.
• ASDs did not show MTG increased activation during mentalizing in social context.
• ASDs did not show NAcc increased activation for processing reward in social context.
• Activation deficits were specific to brain areas involved in social processes.
• Results support both the mind-blindness and the social motivation theories of ASDs.

## 1 Introduction

Autism spectrum disorders (ASDs), including autism, Asperger's syndrome and Pervasive Development Disorder—Not Otherwise Specified (PDD-NOS), are neurodevelopmental conditions characterized by core deficits in social communication skills evident before the age of 3. Interpersonal relationships typically involve effective assessment of one's own state of mind, as well as that of others. The former includes motivational processes that appraise the value to the self of rewards or punishments, real or prospective, as potential consequences of specific social situations. The latter includes mentalizing processes that establish a representation of others' thoughts, desires, goals and beliefs, also known as ‘Theory of Mind (ToM).’ While motivation and mentalizing processes are both engaged simultaneously in real-life social interactions, they are believed to engage selective brain networks (e.g. Adolphs, 2003; Assaf et al., 2009; Chevallier et al., 2012; Dolan, 2002; Frith and Frith, 2003; Phan et al., 2002; Ressler, 2004). Importantly, it has been suggested that abnormal function of the mentalizing and/or the motivation networks in individuals with ASD might underlie their symptoms (e.g. Baron-Cohen, 1995; Chevallier et al., 2012; Dawson et al., 1998, 2005; Frith, 2001; Hill and Frith, 2003; Schultz, 2005). However, to date there is limited and contradictory evidence regarding the specific neural correlates of these networks in individuals with ASDs, and no study has evaluated their *concurrent* function in this population during actual social interaction.

The specialized mentalizing-related network likely involves the temporoparietal junction (TPJ), superior temporal sulcus (STS), temporal pole (TP) and medial prefrontal cortex (MPFC) in typically developed individuals (for review see, Frith and Frith, 2003). Although this network has been mostly studied using explicit ‘off-line’ mentalizing tasks (i.e., tasks where participants are explicitly instructed to *retrospectively* explain the state of mind of other people), implicit ‘on-line’ mentalizing tasks (i.e., tasks where participants are not instructed to be involved in thinking about the other during an on-going interaction, thus mentalizing happens in *real-time* and potentially affects the behavior of the participant him/herself), which emulate real life mentalizing processes better, have shown that the same network is involved in both (Assaf et al., 2009; Gallagher et al., 2002; McCabe et al., 2001; Rilling et al., 2004; Vanderwal et al., 2008). The reward-related motivation network includes a cortical–basal ganglia circuit, with the nucleus accumbens (NAcc), orbitofronal cortex (OFC) and midbrain dopaminergic neurons comprising key regions (for review see Haber and Knutson, 2010). This network is involved in the anticipation and response to both primary and secondary rewards (e.g., food or money, respectively). We have previously shown that the NAcc, as well as other reward-related regions, are also responsive to gains during social competitive interaction, independently of implicit ‘on-line’ mentalizing processes (Admon et al., 2013; Assaf et al., 2009).

There is ample evidence to suggest that mentalizing development and reasoning is impaired in individuals with ASDs (e.g. Baron-Cohen, 1995; Frith, 2001; Hill and Frith, 2003). Indeed, the ‘mind-blindness’ theory, which proposes that mentalizing deficits are the core underlying cause of ASDs (Baron-Cohen, 1995), is one of the leading theories of autism. A few small studies to date have explored brain abnormalities related to explicit ‘off-line’ mentalizing in ASD and results are conflicting, with some showing overactivation and others showing underactivation in individuals with ASDs (Baron-Cohen et al., 1999; Castelli et al., 2002; Happe et al., 1996; Mason et al., 2008; Piggot et al., 2004). To our knowledge, no study to date has explored the potential neural abnormalities of this network during the performance of an implicit ‘on-line’ mentalizing task in ASDs. Studying social function and related neural abnormalities in ASDs with more naturalistic paradigms is of great importance, as there is a discrepancy between performance of individuals with ASDs on explicit social tasks versus performance in more naturalistic implicit social conditions (Klin et al., 2003; Volkmar et al., 2004).

A more recent explanation of ASD symptoms, the social motivation hypothesis, suggests that innately diminished reward valence of social stimuli, such as faces, leads to reduced attention to these stimuli, resulting in both abnormal reward processing and deficits in the social interactions and learning seen in ASD individuals (Chevallier et al., 2012; Dawson et al., 1998, 2005; Schultz, 2005). Notably, compared to mentalizing processes, fewer behavioral and neuroimaging studies have been conducted to support the motivation hypothesis and highlight the potential neural deficits associated with reward-related motivational processes in ASD, and results are conflicting regarding the deficit's specificity to social vs. non-social rewards. Several fMRI studies have explored the reward network in ASD with somewhat contradictory results. Schmitz et al. (2008) and Dichter et al. (2012a) showed hyperactivation of the ACC (Dichter et al., 2012a; Schmitz et al., 2008) and left middle frontal gyrus (Dichter et al., 2012a) in individuals with ASDs in *response* to monetary but not social reward. Scott-Van Zeeland et al. (2010) and Dichter et al. (2012b) found hypoactivation in frontal and striatal regions in response to monetary (Dichter et al., 2012b) and social (Scott-Van Zeeland et al., 2010) rewards. During monetary but not social reward *anticipation*, individuals with ASD showed hypoactivation of NAcc and hyperactivation in right hippocampus (Dichter et al., 2012a). Kohls et al. (2013) found hypoactivation of the ACC and amygdala in response to social and monetary rewards, but only monetary rewards resulted in NAcc hypoactivation. A recent event-related potential (ERP) study (Kohls et al., 2011) demonstrated reduced P3 activity in children with ASD for anticipation of both social and monetary reward cues, but no differences for “consumption” of the rewards. As with neuroimaging studies of mentalizing, to our knowledge no study has explored the reward-motivational network activation during actual social interaction in ASDs. Moreover, no study has explored these two networks' neural function while they are simultaneously engaged in social interaction in ASD subjects.

In the current fMRI study we used an interpersonal competitive game, the Domino task (Admon et al., 2013; Assaf et al., 2009; Hyatt et al., 2012; Kahn et al., 2002), to explore the neural correlates of simultaneous implicit ‘on-line’ mentalizing and reward-related motivation processes in high-functioning ASD subjects compared to matched typically developing controls (TD). Our prior work with the Domino task showed that the implicit ‘on-line’ mentalizing network included the TPJ, STS, MPFC, TP and fusiform gyrus, while the reward-related motivation network subsumed the NAcc, OFC, MFG, SPL, precuneus and cerebellum (Assaf et al., 2009). We hypothesized that compared to TD, individuals with ASD would fail to show increased brain activation in key areas of both mentalizing and motivation networks, when playing the game in social versus non-social contexts, i.e., against a human versus a computer opponent. We further hypothesized that these abnormalities would be specific to these relatively high-level social-cognitive processes, and not be evident in lower-level sensory/motor cortices, during social interaction.

## 2 Methods

### 2.1 Subjects

Thirteen individuals with high-functioning (FSIQ > 70) ASDs (ages 12–24, 10 males) were recruited from the Institute of Living outpatient Child and Adolescent Psychiatry and Community clinics, advertisements in patient and family support groups in the Hartford, CT area, and by word of mouth. The diagnosis of ASDs were confirmed with an observational tool, the Autism Diagnostic Observational Schedule (ADOS, Lord et al., 2000) and by a parental interview, the Autism Diagnostic Interview-Revised (ADI-R, Lord et al., 1994), completed under the supervision of a research-reliable individual (M.A.). Fourteen TD controls (ages 11–23, 11 males) were also recruited to the study by advertisements in area schools and the Olin center website, as well as by word of mouth. ASD was ruled out in TDs using the ADOS (available for 13 of the 14 controls), the Social Communication Questionnaire (SCQ, Rutter et al., 2003) lifetime form (available for 9 controls, including the individual without ADOS), and a detailed health questionnaire. ADOS total scores ranged from 0 to 5 (mean = 1.3 ± 1.7) and SCQ scores ranged from 0 to 3 in this group, well within the normal range. Full scale IQ was assessed with the Vocabulary and Block Design subsets of the Wechsler Adult Intelligence Scale-III (WAIS-III) or Wechsler Intelligence Scale for Children-III (WISC-III) and was available for 12individuals with ASDs and 13 TD. Table 1 summarizes the participants' demographic information. Participants were excluded if they had FSIQ < 70, any MRI contraindication (e.g. in-body metal), present or past neurological disease (including epilepsy), psychiatric illness (in TD group only) or a history of head injury with loss of consciousness for > 10 min. Eight of the 13 individuals with ASDs were medicated when scanned (information was missing for one patient): five received CNS stimulants, three atypical antipsychotic drugs and four SSRI/SNRIs (note that six of the medicated participants were treated with more than one drug). All participants provided written informed consent/minor assent (parents of minor participants signed a consent as well), approved by the Hartford Hospital Institutional Review Board, after a complete description of the study and were compensated for their time.

### 2.2 The Domino Game Task

The Domino task is a two-player competitive computerized game modified from Kahn et al. (2002) and described in detail previously (Assaf et al., 2009). The scanned participant is the player while a computer randomly generates the opponent's responses. However, to test the uniqueness of human-related mentalizing, players are told that they are either playing against a human (the experimenter conducting the scan) or a computer opponent (two runs of each type). Thus, from the participants' perspective, during the human-opponent runs they are playing in an interpersonal competitive context. The game contains a pool of twenty-eight domino-like chips; at the beginning of each game, twelve random domino chips are assigned to the player (shown face up on the screen), four undisclosed chips are assigned to a bank and one randomly chosen opponent's domino chip (constant throughout the game) is shown face-up on the board. The 11 remaining chips from the overall pool are not used in that specific game. Each assigned chip can either match the opponent's chip (have one of the opponent chip's numbers) or not. The player's goal is to dispose of all assigned chips before the game ends (4 min) to receive a monetary bonus ($5/game).

**Fig. 1:**
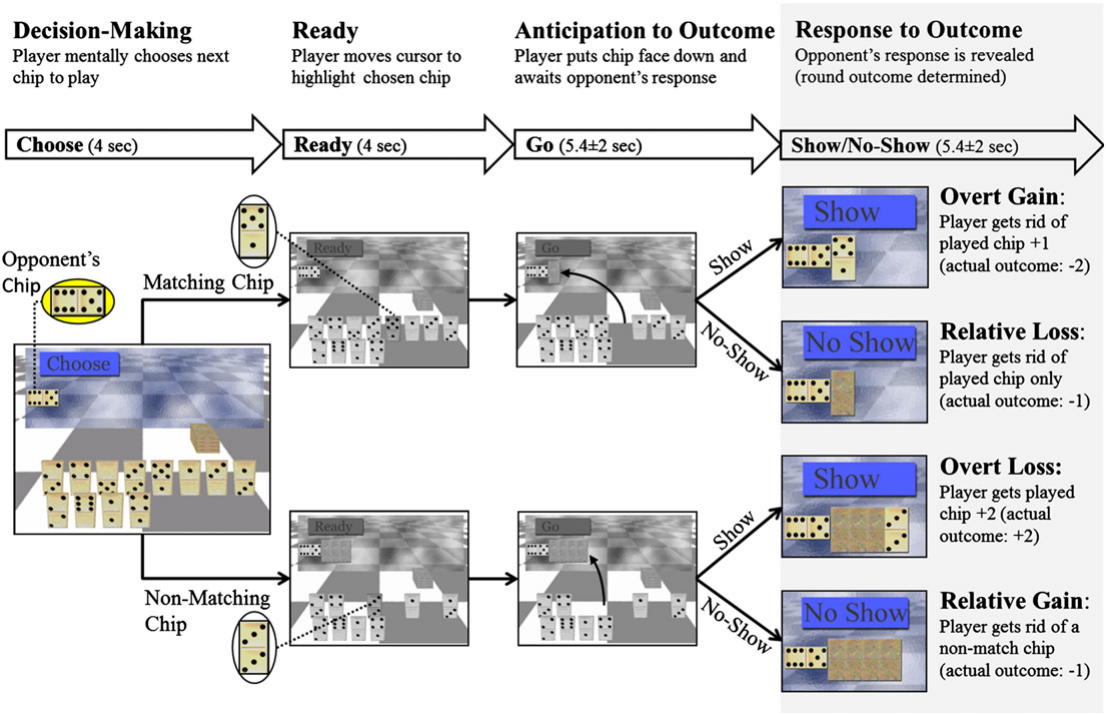
Domino game paradigm. The upper panel describes the 4 intervals that comprise each round of the game: Decision Making, Ready, Anticipation to Outcome and Response to Outcome. The latter is the main focus of this study, thus it is highlighted in gray. The duration of each interval and the command (i.e. event) that starts it are described in the bolded arrows below. The lower panel depicts the Domino Game sequence and corresponding consequences. At the beginning of each game the player (participant scanned) receives 12 playing chips and his/her goal is to dispose of them within 4 min. A constant opponent's chip (in this example 6:5, shown enlarged in the yellow ellipsoid) to which the player matches one chip in each round of the game, is displayed in the upper left corner of the screen throughout the game. Each round starts with the player instructed to decide what chip he/she will play next by the command ‘Choose’ (Decision-making interval). Then the player is instructed to move the cursor to this chip (Ready interval). The chip can either match the opponent's (i.e. have one of the numbers match those on the opponent's chip, upper row, 5:1 in this example) or not (lower row:3:3). After placing the selected chip face down next to the opponent's, he/she awaits the opponent's response (Anticipation of Outcome interval). The opponent can either challenge the player's choice (‘Show’) or not (‘No-Show’). Based on the player's choice and the opponent's response there are four possible consequences for each round (Response to Outcome interval): Show Match (overt gain); No-Show Match (relative loss, as the player could have been rewarded if challenged); Show Non-Match (overt loss) and No-Show Non-Match (relative gain, as the player could have been punished if challenged).

During each round of the game, the player first mentally decides which chip to play next, following the command ‘Choose’; then moves the cursor to it following the command ‘Ready’; next places it face down adjacent to the opponent's chip, following the command ‘Go’; and finally awaits the opponent's response (either 3.4, 5.4 or 7.4 s later) of either ‘Show’ or ‘No-Show’ (see Fig. 1). The ‘Show’ command exposes the player's selected chip, while ‘No-Show’ leaves it unexposed. Following a 5.4 ± 2 second pause after the opponent's response (Response to Outcome interval; see below), the next round begins with the presentation of the ‘Choose’ command.

Based on the player's choice and opponent's response there are four possible consequences per game round, which occur in a well-defined jittered interval, the Response to Outcome interval: (1) Show of Match chip: the choice of a matching chip is exposed and the player is rewarded by disposing of the selected chip plus one additional random chip from the game board. At the end of these trials, players dispose of 2 chips (*overt gain*); (2) Show of Non-Match chip: the player's choice of non-matching chip is exposed and they are punished by receiving back the selected chip plus two additional chips (from the bank or previously played chips, thus not chosen by the player), for a total of 3 chips (i.e. *overt loss*); (3) No-Show of Non-Match chip: a choice of a non-matching chip remains unexposed and only the selected chip is disposed of, so the player is not penalized for a non-matching choice (*relative gain*); and (4) No-Show of Match chip: the choice of a matching chip is not exposed and only the selected matching chip is disposed of, so the player is relatively punished as they could have disposed of an additional chip (*relative loss*). Thus, in the game context, matching chips are considered ‘safe’ moves and non-matching chips are considered ‘risky’ moves or ‘bluffs’, since they are associated with gains and losses of chips, respectively. It is only possible to win and to collect the resulting monetary bonus by occasionally bluffing (i.e. playing a non-matching chip). Importantly, the opponent's chip is constant throughout the game to avoid the possibility of having a matching chip on the board for all rounds of a game which allows the player a game without choosing a non-matching chip. This design ensures the player cannot evade bluffing during most (if not all) games (note that bluffing occurs when the player decides to do so). As in our previous study (Assaf et al., 2009), the player's mentalizing and motivation are tested by contrasting the two possible opponent's responses (‘Show’ vs. ‘No-Show’) and the two possible outcomes (‘Gains’ vs. ‘Losses’), respectively.

Rounds continue until the player wins (by disposing of all of their chips) or loses (when either 240 s have passed or they receive all the chips from the bank and the board back, and there are no more chips they can receive).

Participants played Domino games over 4 scan runs of 10 min each for 12.2 games on average. Participants were told that they were playing against the experimenter for two runs and against the computer, generating random moves, for two. The order of the human and computer opponent runs was counterbalanced across subjects. The experimenter told the participants who they were playing against via headphones immediately before each run began and also made competitive comments regarding the game just played (such as “you really got me this time…”) after human-component runs only.

To ensure that players were engaged in the game and believed that winning was possible, if they did not win during the first run, the first game of the second run was not automated and the experimenter “threw” the game, ensuring that the player won. Sixteen of the 28 players (10 ASDs and 6 TDs) played a non-automated game; these games were excluded from the analysis. Games shorter than one minute were not analyzed as well.

Participants practiced the game outside the scanner prior to scanning. Scanning began when the experimenter was convinced that participants understood the game's rules. A thorough debriefing was carried out immediately after scanning, during which participants were asked about their emotions and strategies while playing the Domino games using open-ended questions and a Likert scale questionnaire where participants rated their responses on a scale of 1(least) to 5 (most) agreement to statements (see examples in Table 2).

### 2.3 Behavioral data analysis

Mixed-effects ANOVAs of Group (ASD vs. TD) by Opponent Type (Human vs. Computer) were conducted to evaluate differences in games played and won and numbers of games shorter than 1 min. Similarly, Likert scale scores were analyzed using a mixed-effects ANOVA using group as a between-subjects variable and opponent type as a within-subjects variable. Follow-up one sample *t*-tests against the middle score of 3 were conducted within group to assess significant agreement or disagreement with the scale's statements.

To characterize players' choices during the game, a Risk Index was defined as the ratio between the number of times a player chose a non-matching chip (only when a choice between non-matching and matching chips was available) to the total number of chips played (again, only when a choice between non-matching and matching chips was available). This index represents an unbiased choice when equal to 0.5 (exactly half of the choices were non-matching choices), a biased choice for matching chips (i.e. playing safer) when < 0.5, or for non-matching chips (i.e. playing riskier) when > 0.5. A mixed-effects ANOVA, using group as a between-subjects variable and opponent type and time (i.e. minutes1 to 4 of the game) as within-subjects variables, was used to assess strategy differences in relation to group, opponent type and game progress in time.

### 2.4 Functional MRI acquisition

Blood oxygenation level dependent (BOLD) data were collected with a T2*-weighted echo planar imaging (EPI) sequence (TR/TE = 1860/27 ms, Flip angle = 70°, Field of view = 22 cm with a 64 × 64 acquisition matrix) using a Siemens Allegra 3 T scanner. Thirty-six contiguous axial functional slices of 3 mm thickness with 1 mm gap were acquired, yielding 3.4 × 3.4 × 4.0 mm voxels. Overall, 330 images were acquired during each run, including 6 ‘dummy’ images at the beginning to allow global image intensity to reach equilibrium, which were excluded from data analysis.

### 2.5 Functional data analysis

Imaging data were analyzed using SPM5 (Wellcome Department of Cognitive Neurology, London, UK). Each individual's data set was realigned to the first ‘non-dummy’ image using the INRIAlign toolbox (A. Roche, INRIA Sophia Antipolis, EPIDAURE Group), spatially normalized to the Montreal Neurological Institute space (Friston et al., 1995) and spatially smoothed with a 9 mm isotropic (FWHM) Gaussian kernel.

As in our previous analyses (Admon et al., 2013; Assaf et al., 2009; Hyatt et al., 2012; Kahn et al., 2002), we defined four intervals of interest to use for fMRI analyses (Fig. 1): The Decision-making interval was defined from the ‘Choose’ command onset to the ‘Ready’ onset, during which players were instructed to decide on their next move without being able to move on the board. The Ready interval was defined from the onset of ‘Ready’ to the onset of ‘Go’. These first two intervals lasted 4 s each. The third interval, Anticipation of Outcome, started after the selected chip was placed face down beside the opponent's chip and ended with the opponent's response. This interval was sorted according to the player's choice of matching or non-matching chips. The fourth interval, Response to Outcome, started after the opponent's response and ended with the next ‘Choose’ onset. Trials were sorted according to the player's choice and the opponent's response to derive the 4 conditions described above (Show Match, Show Non-Match, No-Show Match and No-Show Non-Match). The third and fourth intervals randomly lasted 3.4, 5.4 or 7.4 s each and were thus jittered (Dale and Buckner, 1997).

For every subject, a general linear model (GLM) was estimated with SPM using the ‘Ready’, ‘Anticipation of Outcome’ and ‘Response to Outcome’ intervals as regressors (separately for Human and Computer runs) while the ‘Decision-making’ interval was not modeled to be consistent with previous publications (Admon et al., 2013; Assaf et al., 2009; Kahn et al., 2002). A high-pass filter with a cut-off of 128 s was applied to correct for EPI signal low frequency drift. Next, individual statistical parametric maps of the 4 Response to Outcome interval conditions were calculated for each Opponent type. These maps were entered into two separate whole-brain mixed-effects ANOVAs to delineate the mentalizing and motivation (i.e. reward/gain) networks: (1) A Group (ASD vs. TD) by Opponent Type (Human vs. Computer) by Opponent Response (Show vs. No-Show) ANOVA was calculated to assess group differences in the mentalizing network independently of motivation (gains vs. losses). As described in our previous work (Assaf et al., 2009), we consider both ‘Show’ and ‘No-Show’ conditions to entail mentalizing; however, the ‘Show’ events require more information processing since the player uses new information about the opponent to update his/her representation of the opponent's state of mind (i.e. both strategy and potential next moves). This is due to the fact that the opponent obtains new information about the player during these events (e.g. if the player bluffed or played fairly). From the perspective of the player, the opponent might use this information to change his/her strategy. Thus, the player has to take more information into account when updating his/her representation of the opponent, requiring greater levels of mentalizing. We therefore expected regions related to mentalizing to show differential activation along this parameter in the human-opponent runs and be more prominent in the human- than the computer-opponent runs. Thus, the *mentalizing network* was defined as regions showing greater activation to Show than No-Show conditions in the human-opponent runs as well as a main effect of opponent type such that human was greater than computer (see Assaf et al., 2009) across all participants; (2) A group (ASD vs. TD) by Opponent Type (Human vs. Computer) by Outcome (Gains vs. Losses) mixed-effects ANOVA was calculated to assess group differences in brain activity of motivation processes related to gains (i.e. response to reward) independently of mentalizing (i.e. opponent's response). The *reward-motivation network* was defined as the main effect of Outcome (Gains > Losses) across all subjects. Since we did not expect differences between human- and computer-opponents in responses to gains, the effect of Opponent Type was not integrated into the definition of the motivation network (i.e. main effect of Opponent Type) as for the mentalizing network. For both networks, a mask was created based on the group analyses described above (*q_FDR_ < 0.05, k = 50*), to create regions of interest (ROIs) for follow-up analyses.

To assess group differences within the mentalizing network, we calculated the following contrasts (which are effectively interactions between Group and Opponent Response within the Human-opponent runs): TD (Human Show − Human No-Show) +/− ASD (Human Show − Human No-Show). Group differences within the motivation network were assessed with the contrasts (i.e. interactions): TD (Human Gains − Human Losses) +/− ASD (Human Gains − Human Losses). Note, that for these between-group difference analyses, we used nonparametric permutation-based cluster-level statistical inference. We entered first-level analysis contrasts generated in SPM5 into a second-level analysis using the FSL Randomise v2.1 tool (Oxford University, Oxford UK; http://www.fmrib.ox.ac.uk/fsl/randomise). Since our sample had a wide range of age and IQ scores, we controlled for those parameters in all group analyses (the missing IQ scores of one patient and one control were replaced with the corresponding group average score). Randomise implements a permutation method in which subjects in the design matrix are repeatedly reordered (10,000 iterations) to form the null probability distribution for the maximal cluster mass. Cluster mass is defined as the integral of voxel intensities above the cluster-defining threshold within each cluster, and has been shown to be superior to the use of simple cluster extent (Bullmore et al., 1999; Hayasaka and Nichols, 2004). The cluster-defining threshold for the cluster-level statistics was set at P = 0.05 (uncorrected), and as mentioned above, voxels included in the analysis were mask restricted to those within a given network (e.g., the overall brain network for either the mentalizing or motivation processes). Cluster mass statistics were reported at *P*_cluster_ < 0.05 family-wise error (FWE) rate corrected (i.e., the probability of that cluster mass occurring by chance, within a given brain network, was less than 5%).

In addition, to evaluate the correlations between brain activation related to motivation/mentalizing and the symptom severity of the individuals with ASDs, partial correlation analyses were conducted between the ADOS and SRS scores and the contrasts: Human Show > No-Show and Human Gains > Losses controlling for age and IQ (note that the motivation and mentalizing masks were applied for these analyses as well). Cluster mass threshold was set as described above at *P*_cluster_ < 0.05, FWE corrected.

To assess the specificity of group differences in activation related to human- vs. computer-opponent to the mentalizing and motivation networks, we calculated a 2 × 2 mixed-effects ANOVA of Group × Opponent Type in the Ready interval. This interval includes buttons presses (moving the cursor between the playing chips) and visual input of the game board; thus this analysis explores potential group differences and interaction with opponent type in the motor and visual cortices.

To further evaluate the magnitude of the results, contrast values from individual subjects were extracted for each condition at the points of maximum group results and these values were entered into an ANOVA analysis using SPSS™ (SPSS Inc., Chicago, IL). Note that no new analyses were performed in SPSS.

## 3 Results

### 3.1 Behavioral data

Mixed-effects ANOVAs showed no group differences or group by opponent type interaction in number of games longer than 1 min played and their length, games won or games shorter than 1 min.

Analyses of the Likert scale responses showed that both groups were engaged in the game and wanted to win (Table 2, Q1) and selected their chips intentionally (i.e. not randomly; Table 2, Q2). Mixed-effects ANOVAs of Group by Opponent Type found differences in neither group nor opponent type (Table 2).

In responses to mentalizing statements (e.g. Table 2, Q3), one-sample t-tests showed that TD but not individuals with ASDs took their human-opponent's moves into account when playing and did so more in the human- compared to computer-opponent runs (Human vs. Computer in TD: t(13) = 2.1, p = 0.05). As expected, the repeated measures ANOVA showed a significant main effect of Opponent Type (F(1,21) = 5.7, p = 0.02), such that participants took their human-opponent's moves more into account than computer-opponent's moves. However, there was no significant main effect of group or a group by Opponent Type interaction (F = 0.2, p > 0.01 and F = 0.3, p > 0.1, respectively.

To evaluate the motivational aspect of the game (related to gains and losses), we assessed the players' appraisal (“I was glad when…” or “I was upset when…”) of the various outcomes: overt gains (Table 2, Q4), relative gains (Q5), overt losses (Q6) and relative losses (Q7). As in our previous study (Assaf et al., 2009), TDs' responses to gains (either overt or relative) were significant while responses to losses (overt and relative) were not. The responses of individuals with ASDs were significant to overt gains only. Mixed-effects ANOVAs showed no significant Group or Opponent Type differences or interactions.

**Fig. 2:**
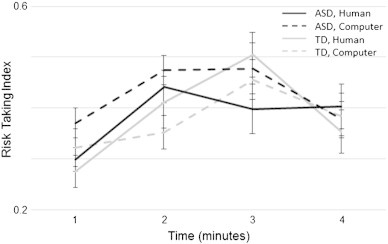
Players' choices as a function of time. Risk-Taking Index (number of non-match choices divided by the total number of non-match and match choices when both are available to the player) for games played against human- (solid lines) and computer- (dashed lines) opponent are plotted for each minute of the game (averaged for all games) for individuals with ASDs (black lines) and healthy controls (gray lines). There was a significant main effect for time (F(1,3) = 6.3, p = 0.002) but not for opponent type or group.

Finally, players' strategy over time as measured by the Risk Taking Index (RTI) was assessed. The average RTI for individuals with ASDs and TD for human- and computer-opponent games were: ASD: 0.39 ± 0.15 and 0.43 ± 0.15, TD: 0.39 ± 0.16 and 0.38 ± 0.16, respectively (no significant Group or Opponent Type effects). A mixed-effects ANOVA of Group by Opponent Type by Time demonstrated a significant main effect of Time (F(1,3) = 6.3, p = 0.002) such that players chose to ‘bluff’ their opponent more towards the middle of a game (minutes 2–3) than at the beginning and end (minutes 1 and 4; see Fig. 2). No significant interactions were found. This is in accord with previous results in healthy individuals (Assaf et al., 2009).

### 3.2 Functional brain data

#### 3.2.1 Mentalizing network

**Fig. 3:**
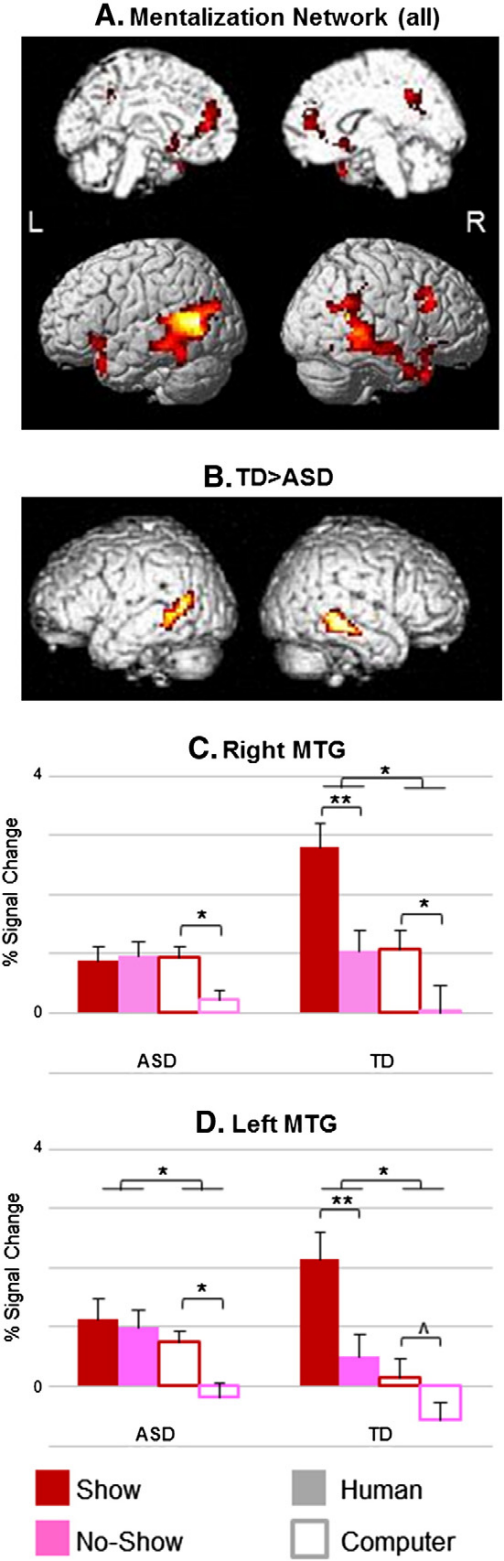
Mentalizing network. Panel A depicts the activation map of a mixed-effects ANOVA showing brain regions with a significant effect of Opponent's Response in the Human-Opponent runs (Show > No-Show) and Opponent Type in all participants (n = 27, q_FDR_ < 0.05). These regions included the temporoparietal junction (TPJ), temporal pole (TP), medial prefrontal cortex (MPFC), posterior cingulate cortex (PCC), ventrolateral prefrontal cortex (VLPFC), fusiform gyrus (FG) and putamen. Panel B shows the map of brain areas exhibiting an interaction between Human Show vs. No-Show events and Group, masked with the mentalizing network as presented in panel A. Bilateral MTGexhibited a significant effect such that controls showed a greater response to Show vs. No-Show compared to individuals with ASDs (left MTG P_cluster_ = 0.03 FWE, right MTG P_cluster_ = 0.04 FWE; for presentation purposes, clusters are shown at a threshold of P < 0.05 uncorrected, k = 20). Percent signal change of the different events in these regions are shown in Panels C & D. **p < 0.001; *p < 0.05; ^p = 0.06; L = left; R = right hemisphere.

As described above and previously (Assaf et al., 2009), to examine the brain areas involved in mentalizing processes regardless of outcome (i.e. motivation) we used the Group by Opponent Type by Opponent Response mixed-effects ANOVA and determined which regions were activated more during Human Show than No-Show events and also more during all human-opponent than computer-opponent events (i.e. main effect of Opponent Type), across all subjects (q_FDR_ < 0.05, k = 50), while controlling for age and IQ. The resulting network is depicted in Fig. 3A and Table 3 and is composed of bilateral temporal poles (TP), temporoparietal junctions (TPJ), including superior temporal sulcus, middle and inferior temporal gyri (STS, MTG and ITG), medial prefrontal cortex (MPFC), posterior cingulate cortex (PCC) and putamen, plus right ventrolateral prefrontal cortex (VLPFC) and fusiform gyrus (FG).

#### 3.2.2 Group differences within the mentalizing network

Interaction analyses revealed group differences in bilateral MTG (Fig. 3B, Table 3), such that TD showed greater activation to the contrast Human Show > No-Show than individuals with ASDs (left and right MTG: *t* = 3.88 and 2.9, respectively; *P*_cluster_ < 0.05, FWE correction), while controlling for age and IQ. Follow-up within subject group ANOVAs demonstrated significantly more activation in Human vs. Computer opponent in left MTG in both groups (ASD: *t* = 1.91, *p* = 0.04; TD: *t* = 3.53, *p* = 0.002) and in right MTG in TD group only (*t* = 3.17, *p* = 0.004). Within group paired *t*-tests demonstrated that individuals with ASDs showed diminished response to the Human Show condition (Fig. 3C and D), such that there were no significant differences between the Show and No-Show events in the Human runs in this group (right and left MTG: *t* = 4.04, *p* = 0.0009 and *t* = 4.29, *p* = 0.0006, respectively). Thus, while controls showed differentiation between Show and No-Show events in the Human Opponent runs as expected (this difference in the Computer Opponent runs showed significance on the right MTG: *t* = 2.62, *p* = 0.01 and only a trend toward significance on the left (*t* = 2.04, *p* = 0.06)), ASD subjects showed no differentiation between the Human Show and No-Show conditions with a much weaker main effect of Opponent Type (due to a differentiation between the Computer Show and No-Show events; see Fig. 3C and D).

#### 3.2.3 Correlation of mentalizing neural activity with patient symptom severity

For the ASD group, we computed partial correlation analyses between the contrast Human Show > No-Show (i.e. Mentalizing) and ADOS and SRS scores within the Mentalizing network (Fig. 3A), while controlling for age and IQ. No cluster survived correction for multiple-comparisons. With more liberal thresholding (p < 0.001, uncorrected, k = 5), one cluster in the MPFC showed significant negative correlation with ADOS Communication subscale (x = − 9, y = 45, z = 9; *r* = − 0.85, p = 0.001; see Supplementary Fig. 1). The correlation pattern suggested that worse symptom severity was associated with lesser activation related to mentalizing processes.

For the ASD group, we computed partial correlation analyses between the contrast Human Show > No-Show (i.e. Mentalizing) and ADOS and SRS scores within the Mentalizing network (Fig. 3A), while controlling for age and IQ. No cluster survived correction for multiple-comparisons. With more liberal thresholding (p < 0.001, uncorrected, k = 5), one cluster in the MPFC showed significant negative correlation with ADOS Communication subscale (x = − 9, y = 45, z = 9; *r* = − 0.85, p = 0.001; see Supplementary Fig. 1). The correlation pattern suggested that worse symptom severity was associated with lesser activation related to mentalizing processes.

#### 3.2.4 Motivation network

**Fig. 4:**
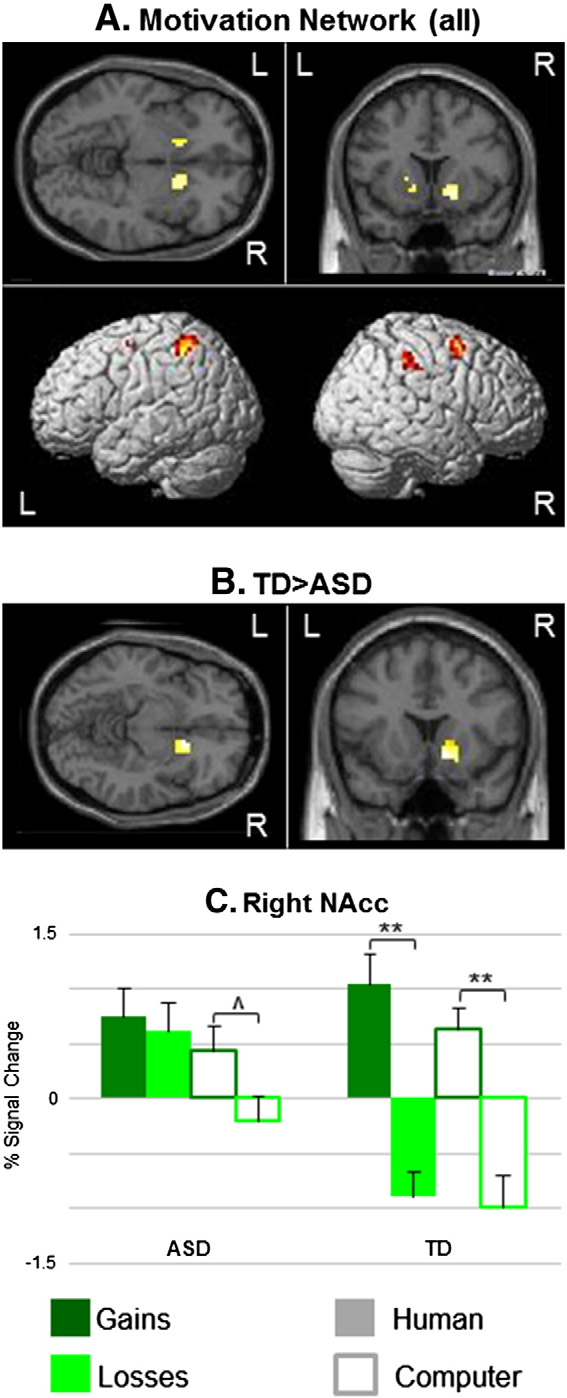
Reward-related Motivation network. Panel (A) shows the activation map of a mixed-effects ANOVA showing brain regions with a significant main effect of Outcome during both human- and computer-opponent runs in all participants (n = 27, q_FDR_ < 0.05). This network includes bilateral NAcc, MFG and SPL. Panel (B) depicts brain regions showing a significant interaction between Outcome and Group masked with regions showing a significant main effect of Outcome as shown in panel A. The right NAcc was the only region showing a significant interaction (P_cluster_ = 0.05 FWE; for presentation purposes cluster is shown at a threshold of P < 0.05 uncorrected, k = 20), such that TD individuals showed significantly more activations for Gains than Losses for both opponents in this region, while individuals with ASDs showed such an effect during the Computer-Opponent games only (panel C). **p < 0.001; *p < 0.05; ^p = 0.06; L = left; R = right hemisphere.

To assess activation related to motivation we used the Group by Opponent Type by Outcome mixed-effects ANOVA and examined the main effect of Outcome (gains vs. losses) across Opponent Type and Group (q_FDR_ < 0.05, k = 50), while controlling for age and IQ. This network included bilateral NAcc, middle frontal gyri (MFG) and superior parietal lobules (SPL), as depicted in Fig. 4A.

#### 3.2.5 Group differences within the motivation network

Interaction analyses demonstrated group differences in right NAcc only (*t* = 3.07, *P*_cluster_ < 0.05, FWE correction; Fig. 4B and Table 3), controlling for age and IQ. While TD showed reward-related activation (gains > losses) in this region overall (*t* = 4.86, *p* = 0.0002) and separately for both Human and Computer runs (*t* = 4.37, *p* = 0.0005 and *t* = 3.94, *p* = 0.001, respectively), individuals with ASDs showed a trend for this effect in Computer runs only (*t* = 1.62; *p* = 0.06); Fig. 4C).

#### 3.2.6 Correlation of motivation neural activity with patient symptom severity

Partial correlation analyses between the contrast Human Gains > Losses and ADOS and SRS scores within the motivation network (Fig. 4A) showed no significant results, even at a low uncorrected threshold (p < 0.01).

#### 3.2.7 Motor and visual cortices

A mixed-effects ANOVA of Group by Opponent Type of the Ready interval events showed a main effect of condition in the leftpre-central gyrus (x = − 45, y = − 30, z = 57; P_FWE_ < 0.05) and primary visual cortices (right: x = 15, y = − 102, z = − 3; left: x = − 24, y = − 102, z = 0; P_FWE_ < 0.05). There was no significant main effect of Group or interaction of Group and Opponent Type in these cortices (see Supplementary Fig. 2).

A mixed-effects ANOVA of Group by Opponent Type of the Ready interval events showed a main effect of condition in the leftpre-central gyrus (x = − 45, y = − 30, z = 57; P_FWE_ < 0.05) and primary visual cortices (right: x = 15, y = − 102, z = − 3; left: x = − 24, y = − 102, z = 0; P_FWE_ < 0.05). There was no significant main effect of Group or interaction of Group and Opponent Type in these cortices (see Supplementary Fig. 2).

## 4 Discussion

We used an *apriori* defined interval during a competitive, two-player fMRI game, to delineate the neural function of brain areas selectively involved in implicit ‘on-line’ mentalizing and in reward-related motivation processes in high-functioning individuals with ASDs compared to healthy controls. Results demonstrated deficits in the MTG for mentalizing, and in the NAcc for motivation, but not in motor and visual areas in the ASD group. These functional deficits were observed only when the individuals with ASDs were involved in a social interaction, representing failure of the MTG and NAcc to increase activity in these individuals in a social context compared to a perceptually identical non-social situation. Thus, when playing in a non-social context (i.e. against a computer-opponent), individuals with ASDs showed a pattern of brain activations similar to controls, but they failed to show the anticipated increase in activation in a social context (i.e. when playing against what they believed was a human-opponent) in the MTG and NAcc. Notably, individuals with ASDs showed comparable understanding of and performance on the Domino task — they played and won similar numbers of games, showed similar risk taking behavior over time, were as eager to play the game and chose chips to play intentionally as controls. No differences were found between the groups on their responses on a post-scan debriefing to mentalizing and motivation statements; however, for the mentalizing statements (e.g. Table 2, Q3) participants with ASDs did not report taking their human- and computer-opponent's moves into account when playing, as did the controls. Below we discuss our results pertaining to the activation of the mentalizing and motivation networks in individuals with ASDs and controls.

### 4.1 The mentalizing network

We showed previously that while playing the Domino games, healthy individuals engage in mentalizing processes and activate a neural network known to be involved in mentalizing (Assaf et al., 2009). Consistent with these previous results, TD individuals in the current study indicated they were taking into account their opponent's responses before deciding on their next move, significantly more so for human- than computer-opponent games. Conversely, although group differences were not significant, the responses of individuals with ASDs on the Likert scale to mentalizing statements (e.g. Q3 in Table 2) were not significantly different from the neutral score of 3 for either of the opponents and there was no difference between the opponent types, indicating that they were not consciously thinking about their opponent, and thus they might not have fully engaged the mentalizing network while playing.

The implicit ‘on-line’ mentalizing-related areas were defined as areas showing the combined effect of Opponent's Response/Mentalizing in Human-Opponent (‘Show’ vs. ‘No-Show’) and Opponent Type (Human vs. Computer) regardless of the Motivation effect, in all participants. This network included the TPJ (STS, MTG and ITS), TP, MPFC, PCC, FG, VLPFC and putamen. Except for the 2 latter areas, these areas were demonstrated in our previous work (Assaf et al., 2009) and others' explicit ‘off-line’ and implicit ‘on-line’ mentalizing neuroimaging studies (e.g. Assaf et al., 2009; Castelli et al., 2000; Frith and Frith, 2003; Gallagher et al., 2000, 2002; McCabe et al., 2001; Rilling et al., 2004; Vanderwal et al., 2008; Vogeley et al., 2001). Contrary to the current results, we previously showed that VLPFC activation was sensitive to mentalizing, but not to opponent type, and concluded that its activation is related to general top-down regulation that is not specific to social processes (Assaf et al., 2009). However, the current results as well as other reports support a more specific involvement in social-cognitive processes, such as trustworthiness (Pinkham et al., 2008) and mentalizing (e.g. Kobayashi et al., 2007). To the best of our knowledge, the putamen has not previously been implicated in mentalizing. It is possible that its activation in our study is related to the general reward associated with social interaction (Krach et al., 2010). More work should be done to confirm this result.

As mentioned above, individuals with ASDs showed abnormal mentalizing activation in bilateral MTG. In both areas, group differences were the result of diminished response to the Show events, which we suggest entails a higher mentalizing load than the No-Show events, during the Human-Opponent runs only in the ASD group. Thus, individuals with ASDs show no differentiation between the Show and No-Show conditions when playing against a human opponent. BOLD responses during the Computer-Opponent runs in this group were similar to those of controls. In addition, albeit at a more relaxed statistical threshold, the activation of the MPFC correlated with the symptom severity of individuals with ASDs as measured by the ADOS Communication subscale score, such that individuals with more severe deficits showed less mentalizing-related activation in this region (note that medication at time of scan did not seem to drive the results; see Supplementary Fig. 1).

As mentioned above, individuals with ASDs showed abnormal mentalizing activation in bilateral MTG. In both areas, group differences were the result of diminished response to the Show events, which we suggest entails a higher mentalizing load than the No-Show events, during the Human-Opponent runs only in the ASD group. Thus, individuals with ASDs show no differentiation between the Show and No-Show conditions when playing against a human opponent. BOLD responses during the Computer-Opponent runs in this group were similar to those of controls. In addition, albeit at a more relaxed statistical threshold, the activation of the MPFC correlated with the symptom severity of individuals with ASDs as measured by the ADOS Communication subscale score, such that individuals with more severe deficits showed less mentalizing-related activation in this region (note that medication at time of scan did not seem to drive the results; see Supplementary Fig. 1).

We point out that although TPJ is more traditionally considered a core mentalizing region than MTG/STS and thus group differences might be expected there, a recent review showed that these regions are almost equally activated by mentalizing tasks in general, and STS/MTG is activated more often in tasks associated with intentions (Carrington and Bailey, 2009). In addition, other mentalizing studies implicate this region in ASDs. As mentioned in the introduction, only a handful of neuroimaging studies have explored the mentalizing network in individuals with ASDs, most using explicit ‘off-line’ mentalizing tasks, with conflicting results. Using reading comprehension tasks of theory of mind (ToM) stories, where participants are instructed to infer the intentions of other people, Happe et al. (1996) showed hypoactivation of left PFC in BA 8/9 and increased activation in an adjacent area, BA 9/10, in 5 adults with Asperger's syndrome. Conversely, Mason et al. (2008) showed hyperactivation of the right temporo-parietal junction, including MTG clusters overlapping our cluster showing mentalizing group differences, in 18 high-functioning individuals with ASDs during different types of inferences, including emotional and intentional ToM processes (see Supplementary Fig. 3). Using tasks that require inferring the emotions of others by expressions of the face or eyes only, Piggot et al. (2004) showed hypoactivation of the fusiform gyrus in 14 adolescents with high-functioning ASD, while Baron-Cohen et al. (1999) showed hypoactivation of the amygdala but not STS and PFC in six adults with autism. Castelli et al. (2002) used the Social Attribution fMRI task, where participants were asked to describe short animations of shapes moving in either random, goal-directed or intentionally-directed fashions (the latter involved mentalizing). Importantly, this task is closest to ours conceptually, as it also employs ‘on-line’ mentalizing (albeit not implicitly). They showed hypoactivation in individuals with ASDs in several areas of the mentalizing network, including temporo-parietal junction and superior temporal sulcus, areas adjacent to the left MTG cluster showing hypoactivation in the ASD group in our study (Supplementary Fig. 3). Our results show specific deficits in the MTG during mentalizing while individuals with ASDs are competing with what they believe is another person, thinking about his/her goals and intentions, and updating their behavior accordingly (implicit ‘on-line’ mentalizing). These results further support the ‘mind-blindness’ theory (e.g. Baron-Cohen, 1995), which suggests that deficits in the mentalizing network are the basis of ASD psychopathology, pointing to the MTG and MPFC as the areas affected.

We point out that although TPJ is more traditionally considered a core mentalizing region than MTG/STS and thus group differences might be expected there, a recent review showed that these regions are almost equally activated by mentalizing tasks in general, and STS/MTG is activated more often in tasks associated with intentions (Carrington and Bailey, 2009). In addition, other mentalizing studies implicate this region in ASDs. As mentioned in the introduction, only a handful of neuroimaging studies have explored the mentalizing network in individuals with ASDs, most using explicit ‘off-line’ mentalizing tasks, with conflicting results. Using reading comprehension tasks of theory of mind (ToM) stories, where participants are instructed to infer the intentions of other people, Happe et al. (1996) showed hypoactivation of left PFC in BA 8/9 and increased activation in an adjacent area, BA 9/10, in 5 adults with Asperger's syndrome. Conversely, Mason et al. (2008) showed hyperactivation of the right temporo-parietal junction, including MTG clusters overlapping our cluster showing mentalizing group differences, in 18 high-functioning individuals with ASDs during different types of inferences, including emotional and intentional ToM processes (see Supplementary Fig. 3). Using tasks that require inferring the emotions of others by expressions of the face or eyes only, Piggot et al. (2004) showed hypoactivation of the fusiform gyrus in 14 adolescents with high-functioning ASD, while Baron-Cohen et al. (1999) showed hypoactivation of the amygdala but not STS and PFC in six adults with autism. Castelli et al. (2002) used the Social Attribution fMRI task, where participants were asked to describe short animations of shapes moving in either random, goal-directed or intentionally-directed fashions (the latter involved mentalizing). Importantly, this task is closest to ours conceptually, as it also employs ‘on-line’ mentalizing (albeit not implicitly). They showed hypoactivation in individuals with ASDs in several areas of the mentalizing network, including temporo-parietal junction and superior temporal sulcus, areas adjacent to the left MTG cluster showing hypoactivation in the ASD group in our study (Supplementary Fig. 3). Our results show specific deficits in the MTG during mentalizing while individuals with ASDs are competing with what they believe is another person, thinking about his/her goals and intentions, and updating their behavior accordingly (implicit ‘on-line’ mentalizing). These results further support the ‘mind-blindness’ theory (e.g. Baron-Cohen, 1995), which suggests that deficits in the mentalizing network are the basis of ASD psychopathology, pointing to the MTG and MPFC as the areas affected.

### 4.2 The motivation network

As in previous results, behavioral data showed that the Domino game is sensitive to the valence of gains (i.e. reward), either overt or relative, but not to losses (i.e. punishment) in healthy individuals. This was true in the ASD group for overt gains only, although no significant between-group differences were found. The reward-related motivation network was defined as areas showing more activation to gains than losses in both human- and computer-opponent runs in all participants, independently of the mentalizing effect. This network included bilateral NAcc, MFG and SPL (post-central gyrus). These results are in agreement with our previous study in healthy individuals (Assaf et al., 2009) and with other research on the reward network (for review see Haber and Knutson, 2010).

Group differences in the Motivation network were detected only in the right NAcc. As for the Mentalizing network, these differences resulted from failure of NAcc activation to increase in response to gains during the Human-Opponent runs, leading to lack of differentiation between the Gains and Losses events in these runs in individuals with ASDs. Activations related to Gains and Losses during the Computer-Opponent runs were similar to those of controls (albeit statistically not as strong).

To our knowledge, only five other studies to date have explored the reward network activation in individuals with ASDs; four showed decreased patient activation in NAcc. Dichter et al. (2012b) used a monetary and patient-salient objects modified incentive delay fMRI task to assess the neural correlates of adults with high-functioning ASD compared to TD during anticipation and response to the two reward types. They demonstrated an interaction between group and reward type during reward *anticipation* in bilateral NAcc, such that participants with ASD shad hypoactivation to monetary but not to object rewards. In a more recent study from this group (Dichter et al., 2012a), using a similar fMRI task but replacing the ASD-salient object with face images to represent social rewards, individuals with ASDs also showed hypoactivation of the right NAcc during monetary – but not social – reward *anticipation*. Kohls et al. (2013) also showed hypoactivation of the NAcc in ASDs, again during monetary but not social reward processing, using similar task. Importantly, the latter authors questioned the use of static face images as social reward stimuli. Conversely, Scott-Van Zeeland et al. (2010) used an fMRI reward learning task with either social (i.e. happy or sad faces) or monetary rewards with high-functioning ASD adolescent boys. They demonstrated hypoactivation of fronto-striatal regions, including the NAcc, in *response* to social rewards and social, but not monetary, reward learning.

Our results further suggest a deficit in NAcc activation during *response* to rewards. This deficit is specific to a social context of a competitive interaction with a perceived human vs. identical non-social interaction with a computer, emphasizing the deficits in processing rewards in a social context vs. social rewards per se (e.g. faces), as this hypothesis has been interpreted and tested in the previous neuroimaging studies (e.g. Dichter et al., 2012a; Scott-Van Zeeland et al., 2010). Importantly, our results are in accord with the social motivation hypothesis (Chevallier et al., 2012; Dawson et al., 1998, 2005; Schultz, 2005); however, given our similar results related to mentalizing processes and their neural correlates, and the lack of similar effects in motor and visual cortices, this social deficit does not seem to be specific to motivation processes and can be seen in other, related *social-cognitive processes* during inter-personal interaction. Our study is not designed to provide evidence for causality between social motivation and cognition processes (i.e. mentalizing) as hypothesized by others (e.g. Chevallier et al., 2012). Taken together, these results can explain the neural basis of the observed decreased response of individuals with ASDs to human/social reinforcement (Freitag, 1970; Garretson et al., 1990; Geurts et al., 2008) and have implications for treatment development. They also emphasize the critical importance of testing and treating individuals with ASDs in a social context (i.e. while being involved with other people) to reliably assess their abilities and treatment efficacy in ‘real-life’ situations.

### 4.3 The executive dysfunction and weak central coherence theories of autism

The mentalizing and motivation theories are only two of several existing models that attempt to explain the symptoms of ASDs. Two other leading theories are the executive dysfunction (EF) and weak central coherence (WCC) hypotheses (for review see Rajendran and Mitchell, 2007). Both theories suggest a more general-domain deficit. The EF theory suggests that impairments in executive functions underlie the deficiencies seen in ASDs, while the WCC theory suggests that individuals with ASDs fail to process information in a global, coherent way but rather they are focused on details. We note that executive functions and central (or global) coherence processing are most likely utilized when playing Domino to maintain information on the game's rules and progress and to decide on their own moves, and might be required for mentalizing and motivation processes. However, because our design does not manipulate players' behavior on the EF and central coherence processing domains, we cannot refer to related behavioral or neurological impairments in ASDs. Having said that, the fact that individuals with ASD played similarly to controls might imply intact EF processing. Future research should explore this in more depth.

### 4.4 Study limitations

We note several limitations of our study. First, we report results from only 13 individuals with the diagnosis of ASDs; and although our major findings were statistically reliable, replication of our results in a larger sample would be helpful. Second, most of these individuals (8/13) were medicated at the time of scan, and thus we cannot conclude with confidence that results are not confounded by medication effects (although, as can be seen in Supplementary Fig. 1, neither of the 2 ASD groups (medicated vs. non-medicated) is driving the correlation of symptom severity and MPFC activation; we lack the power to repeat analyses with medicated/unmediated individuals only). Third, as mentioned above, it is possible that diminished neural response to motivational processes in the ASD group is not reward-specific and can also be evident in negative-valence (i.e. punishment) motivational events. However, since the Domino task does not reliably activate brain areas involved in response to punishment (probably due to low valence of punishment events in the context of this game, Assaf et al., 2009), punishment-related neural networks could not be assessed in this study. Future studies should investigate this network in individuals with ASDs, preferably in relation to reward processing. Finally, this manuscript is focused on the Response to Outcome interval, which includes response but not anticipation to rewards. Importantly, the anticipation interval in the Domino task includes the potential of experiencing gains or losses (overt or relative), thus contrary to other reward tasks such as the monetary incentive delay task (e.g. Knutson et al., 2000; Kohls et al., 2013), brain activity related to reward anticipation is difficult to distinguish from punishment anticipation. Follow-up studies should be designed a priori to explore brain activity related to reward anticipation during a social interactive task.

We note several limitations of our study. First, we report results from only 13 individuals with the diagnosis of ASDs; and although our major findings were statistically reliable, replication of our results in a larger sample would be helpful. Second, most of these individuals (8/13) were medicated at the time of scan, and thus we cannot conclude with confidence that results are not confounded by medication effects (although, as can be seen in Supplementary Fig. 1, neither of the 2 ASD groups (medicated vs. non-medicated) is driving the correlation of symptom severity and MPFC activation; we lack the power to repeat analyses with medicated/unmediated individuals only). Third, as mentioned above, it is possible that diminished neural response to motivational processes in the ASD group is not reward-specific and can also be evident in negative-valence (i.e. punishment) motivational events. However, since the Domino task does not reliably activate brain areas involved in response to punishment (probably due to low valence of punishment events in the context of this game, Assaf et al., 2009), punishment-related neural networks could not be assessed in this study. Future studies should investigate this network in individuals with ASDs, preferably in relation to reward processing. Finally, this manuscript is focused on the Response to Outcome interval, which includes response but not anticipation to rewards. Importantly, the anticipation interval in the Domino task includes the potential of experiencing gains or losses (overt or relative), thus contrary to other reward tasks such as the monetary incentive delay task (e.g. Knutson et al., 2000; Kohls et al., 2013), brain activity related to reward anticipation is difficult to distinguish from punishment anticipation. Follow-up studies should be designed a priori to explore brain activity related to reward anticipation during a social interactive task.

## 5 Conclusions

Using an fMRI competitive game, the Domino paradigm, we showed that individuals with high-functioning ASDs failed to show the typical increased activation in MTG during mentalizing processing and in the NAcc during reward processing, in a social context (i.e. when apparently playing against another human), compared to a perceptually identical non-social situation (i.e. when playing against a computer) where they show typical activations. These activation deficits were specific to brain areas involved in social processes and were not demonstrated in motor and visual cortices, thus they are in accord with both the mind-blindness and the social motivation theories of ASDs, emphasizing the importance of the social (vs. non-social) context of the experimental design (vs. the specific social-cognitive process) when exploring social-motivation and mentalizing. Individuals with ASDs are known to have reduced motivation in social contexts, with potential implication for treatment methods/response (Freitag, 1970; Garretson et al., 1990; Geurts et al., 2008); our study is the first to demonstrate the neural correlates of this phenomenon with a natural, interactive task.

The following are the supplementary data related to this article.

Supplementary Fig. 1 Correlation between Mentalizing contrast in Human runs (Show > No-Show) and ADOS Communication scores in individuals with ASDs. As shown in panel (A), only the MPFC showed significant correlations within the Mentalizing network as outlined in Fig. 3A (p < 0.01, k = 5) such that BOLD signal negatively correlated with ADOS Communication subscale scores (panel B; r = -0.85, p = 0.001). Individuals receiving treatment at the time of the scan are indicated by filled diamonds, individuals not receiving treatment in empty diamonds and the participant with unknown medication status in a filled gray square.

Supplementary Fig. 2 Motor and visual cortices activation during the Ready interval. The upper panel shows activation map of a mixed-effects ANOVA of Group by Opponent Type of the Ready interval events, showing a main effect of condition in the left pre-central gyrus and primary visual cortices (PFWE < 0.05). As can be seen in the lower 3 panels, there was neither significant main effect of Group or Opponent Type nor interaction of Group and Opponent Type in these areas.

Supplementary Fig. 3 Comparison with previous studies. This figure compares the MTG areas showing group differences in our study (red clusters) to previously reported differences in TPJ/STS/MTG activation related to mentalizing processes in individuals with ASDs. Castelli et al. (2002) demonstrated hypoactivation of bilateral TPJ/STS in individuals with ASD compared to healthy controls when participants looked at animated shaped “interacting” vs. moving randomly (green). Mason et al. (2008) showed hyperactivation of STS/MTG in the ASD group when individuals performed tasks of intentional (blue), emotional and physical inferences. Note that the green and blue indicators are spheres with 6 mm radius around the reported peak activation and not the whole clusters presented in these studies.

Supplementary data to this article can be found online at http://dx.doi.org/10.1016/j.nicl.2013.09.005.

## Figures and Tables

**Table 1:** Demographic, symptoms assessment and behavioral information. (Mean scores ± standard deviation).

	ASD (n = 13)	TD (n = 14)	Group statistics	p
Age (years) (range)	17.5 ± 3.3 (12–24)	17.4 ± 3.6 (11–23)	t(25) = 0.1	n.s.
Gender (M/F)	10/3	11/3	χ^2^(1) = 0.01	n.s.
Handedness (R/L)	11/2	13/1	χ^2^(1) = 0.4	n.s.
Race (W/B/O)	12/0/1	14/0/0	χ^2^(3) = 1.1	n.s.
FSIQ (n = 12/13) (range)	110.9 ± 21.2 (71–135)	122.3 ± 12.8 (100–144)	t(23) = − 1.6	n.s.
ADOS-Total (n = 13/13)	13.7 ± 3.9	1.3 ± 1.7	t(24) = 10.4	< 0.0001
ADOS-Communication (n = 14/13)	4.5 ± 1.3	0.9 ± 1.4	t(24) = 6.8	< 0.0001
ADOS-Social (n = 14/13)	9.2 ± 3.3	0.4 ± 0.9	t(24) = 9.1	< 0.0001
Games Played: Human Opponent	4.8 ± 0.7	5.1 ± 0.9	F(1) = 1.08	n.s.
Computer Opponent	5.3 ± 1.2	5.2 ± 0.8		
Duration of Games (minutes): Human	3.6 ± 0.4	3.5 ± 0.5	F(1) = 1.89	n.s.
Computer	3.4 ± 0.1	3.4 ± 0.4		
Games Won: Human	0.9 ± 0.9	1.2 ± 1.2	F(1) = 0.05	n.s.
Computer	1.6 ± 0.9	1.2 ± 0.8		

**Table 2:** Participants' responses to post-scan debriefing.

Question		ASD		TD		Group (GR) × Opponent Type (OT) ANOVA		
		Human	Computer	Human	Computer	GR ME	OT ME	Interaction
Q1	I did everything I could to win the game	4.5 ± 1.3**	4.5 ± 1.3**	4.3 ± 1.0**	4.0 ± 1.0**	n.s.	n.s.	n.s.
Q2	I only played the chips after choosing them first	4.2 ± 1.3*	4.2 ± 1.1*	3.8 ± 1.0*	4.2 ± 1.1**	n.s.	n.s.	n.s.
Q3	I took my opponent's last moves into account before deciding which chip to play next (M)	3.9 ± 1.7	3.7 ± 1.6	4.2 ± 0.7**	3.8 ± 1.0*	n.s.	F = 5.7, p = 0.02	n.s.
Q4	I felt glad when a matching chip was challenged (OG)	4.4 ± 1.3*	4.2 ± 1.4*	4.2 ± 0.9**	4.2 ± 0.9**	n.s.	n.s.	n.s.
Q5	I felt glad when a non-matching chip was not challenged (RG)	3.7 ± 1.7	3.6 ± 1.7	4.1 ± 1.2**	4.1 ± 1.2**	n.s.	n.s.	n.s.
Q6	I felt upset when a non-matching chip was challenged (OL)	2.5 ± 1.6	2.7 ± 1.6	2.7 ± 1.6	2.9 ± 1.0	n.s.	n.s.	n.s.
Q7	I felt upset when a matching chip was not challenged (RL)	3.8 ± 1.4	3.7 ± 1.4	3.3 ± 1.1	3.1 ± 1.0	n.s.	n.s.	n.s.

* p < 0.05, ** p < 0.005 for one-sample *t*-test, testing difference from the middle score of 3. G = Group (ASD, TD); OT = Opponent Type (Human, Computer); ME = Main effect; M = Mentalizing question; OG = Overt Gains; RG = Relative Gains; OL = Overt Losses; RL = Relative Losses.

**Table 3:** Brain regions activated during the Response to Outcome interval.

Anatomic location of maximum activation	MNI coordinates			T score
	x	y	z	
*Mentalizing Network*				
Human Show > No-Show masked with Human > Computer (all subjects)				
L TPJ (ITS/MTG/STS)	− 54	− 60	9	6.66
R TPJ (ITS/MTG/STS)	57	− 48	12	6.40
L TP	− 39	18	− 30	3.40
R TP	36	18	− 39	3.52
R FG	45	− 45	− 18	5.26
MPFC	0	42	12	3.75
PCC	9	− 45	39	3.99
R VLPFC	51	15	36	5.74
L Putamen	− 21	9	− 15	4.00
R Putamen	15	12	− 6	3.18
Healthy Controls > ASD individuals				
L MTG	− 60	− 45	− 3	2.54
R MTG	57	− 30	− 12	3.20

*Reward-related Motivation Network*				
Gains > Losses (all subjects)				
L NAcc	− 12	15	− 3	4.19
R NAcc	18	12	− 9	5.10
L MFG (BA 6)	− 30	− 6	57	4.55
R MFG (BA 6)	33	3	57	5.33
L SPL	− 39	− 48	57	5.18
R SPL	42	− 30	42	5.10
Healthy Controls > ASD individuals				
R NAcc	15	15	− 9	3.07

BA, Brodmann region; FG, fusiform gyrus; ITS, inferior temporal sulcus; MFG, middle frontal gyrus; MPFC, medial prefrontal cortex; MTG, middle temporal gyrus; NAcc, nucleus accumbens; OFC, orbitofrontal cortex; PCC, posterior cingulate cortex; SPL, superior parietal lobule; STS, superior temporal sulcus; TP, temporal pole; TPJ, temporoparietal junction; VLPFC, ventrolateral prefrontal cortex; L, left; R, right.
