# Supplementary material for: Mentalizing and motivation neural function during social interactions in autism spectrum disorders
Source: Neuroimage Clin. 2013 Sep 19;3:321–31. doi: 10.1016/j.nicl.2013.09.005 (PMC3815022; doi:10.1016/j.nicl.2013.09.005)

**Supplementary Figure 1.**Correlation between Mentalizing contrast in Human runs (Show>No-Show) and ADOS Communication scores in individuals with ASDs. As shown in panel (**A)**, only the MPFC showed significant correlations within the Mentalizing network as outlined in Figure 3.A (p<0.01, k=5) such that BOLD signal negatively correlated with ADOS Communication subscale scores (panel **B**; r=-0.85, p=0.001). Individuals receiving treatment at the time of the scan are indicated by filled diamonds, individuals not receiving treatment in empty diamonds and the participant with unknown medication status in a filled gray square.


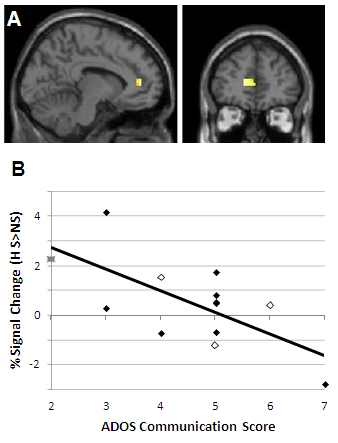

Supplement: Supplementary Fig. 1 — Correlation between Mentalizing contrast in Human runs (Show > No-Show) and ADOS Communication scores in individuals with ASDs. As shown in panel (A), only the MPFC showed significant correlations within the Mentalizing network as outlined in Fig. 3A (p < 0.01, k = 5) such that BOLD signal negatively correlated with ADOS Communication subscale scores (panel B; r = -0.85, p = 0.001). Individuals receiving treatment at the time of the scan are indicated by filled diamonds, individuals not receiving treatment in empty diamonds and the participant with unknown medication status in a filled gray square. [file mmc1.docx]
