# Supplementary material for: Mentalizing and motivation neural function during social interactions in autism spectrum disorders
Source: Neuroimage Clin. 2013 Sep 19;3:321–31. doi: 10.1016/j.nicl.2013.09.005 (PMC3815022; doi:10.1016/j.nicl.2013.09.005)

**Supplementary Figure 2.** Motor and Visual cortices activation during the Ready interval. The upper panel shows activation map of a mixed-effects ANOVA of Group by Opponent Type of the Ready interval events, showing a main effect of condition in the left pre-central gyrus and primary visual cortices (P_FWE_<0.05). As can be seen in the lower 3 panels, there was neither significant main effect of Group or Opponent Type nor interaction of Group and Opponent Type in these areas.


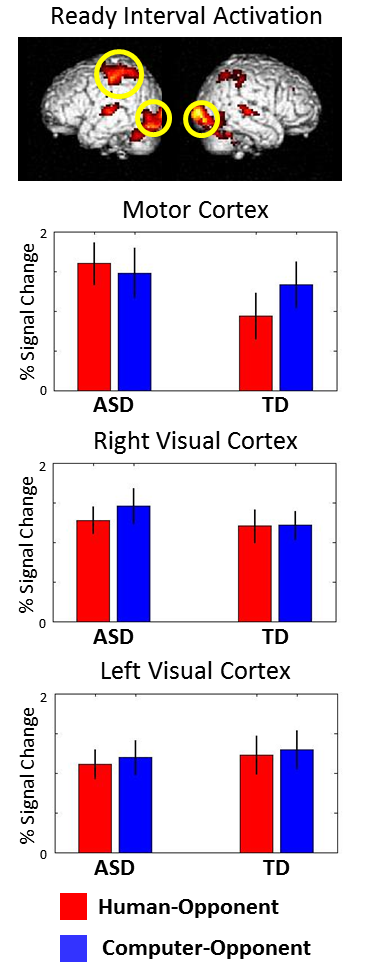

Supplement: Supplementary Fig. 2 — Motor and visual cortices activation during the Ready interval. The upper panel shows activation map of a mixed-effects ANOVA of Group by Opponent Type of the Ready interval events, showing a main effect of condition in the left pre-central gyrus and primary visual cortices (PFWE < 0.05). As can be seen in the lower 3 panels, there was neither significant main effect of Group or Opponent Type nor interaction of Group and Opponent Type in these areas. [file mmc2.docx]
