# Supplementary material for: Mentalizing and motivation neural function during social interactions in autism spectrum disorders
Source: Neuroimage Clin. 2013 Sep 19;3:321–31. doi: 10.1016/j.nicl.2013.09.005 (PMC3815022; doi:10.1016/j.nicl.2013.09.005)

**Supplementary Figure 3.** Comparison with previous studies. This figure compares the MTG areas showing group differences in our study (red clusters) to previously reported differences in TPJ/STS/MTG activation related to mentalizing processes in individuals with ASDs. Castelli et al. (2002) demonstrated hypoactivation of bilateral TPJ/STS in individuals with ASD compared to healthy controls when participants looked at animated shaped "interacting" vs. moving randomly (green). Mason et al. (2008) showed hyperactivation of STS/MTG in the ASD group when individuals performed tasks of intentional (blue), emotional and physical inferences. Note that the green and blue indicators are spheres with 6 mm radius around the reported peak activation and not the whole clusters presented in these studies.


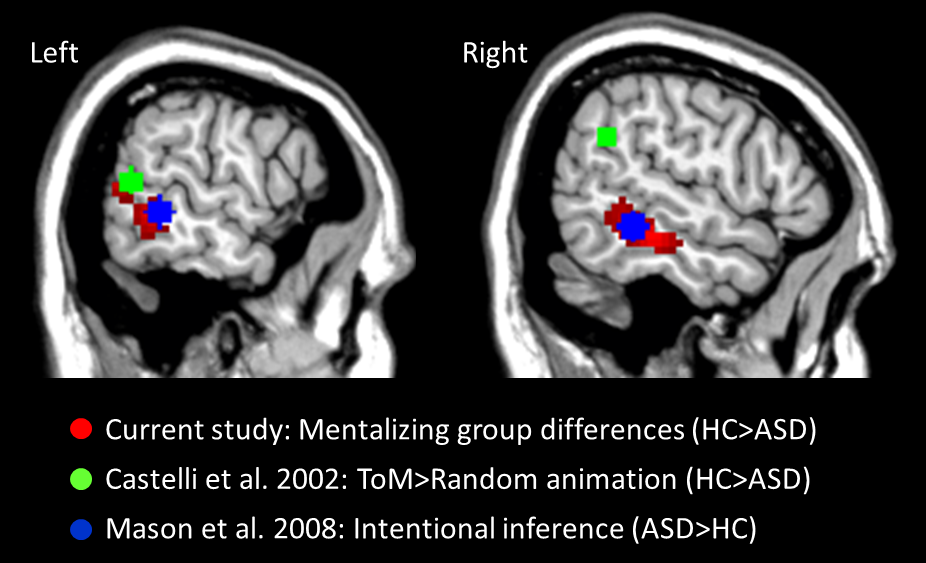

Supplement: Supplementary Fig. 3 — Comparison with previous studies. This figure compares the MTG areas showing group differences in our study (red clusters) to previously reported differences in TPJ/STS/MTG activation related to mentalizing processes in individuals with ASDs. Castelli et al. (2002) demonstrated hypoactivation of bilateral TPJ/STS in individuals with ASD compared to healthy controls when participants looked at animated shaped “interacting” vs. moving randomly (green). Mason et al. (2008) showed hyperactivation of STS/MTG in the ASD group when individuals performed tasks of intentional (blue), emotional and physical inferences. Note that the green and blue indicators are spheres with 6 mm radius around the reported peak activation and not the whole clusters presented in these studies. [file mmc3.docx]
